# Neovascular Glaucoma from Ocular Ischemic Syndrome Treated with Serial Monthly Intravitreal Bevacizumab and Panretinal Photocoagulation: A Case Report

**DOI:** 10.1155/2022/4959522

**Published:** 2022-07-28

**Authors:** Hassaan Asif, Zhuangjun Si, Steven Quan, Pathik Amin, David Dao, Lincoln Shaw, Dimitra Skondra, Mary Qiu

**Affiliations:** Department of Ophthalmology and Visual Science, University of Chicago, Chicago, Illinois, USA

## Abstract

**Purpose:**

To describe a case of open-angle neovascular glaucoma (NVG) secondary to ocular ischemic syndrome (OIS) treated with a planned series of 6 monthly anti-VEGF injections with interspersed panretinal photocoagulation (PRP) sessions. We term this treatment protocol the Salvaging Conventional Outflow Pathway in Neovascular Glaucoma (SCOPING) Protocol, and this is our (MQ and DS) standard of care for all NVG patients presenting with partially or completely open angles.

**Case:**

A 66-year-old man's right eye had a visual acuity of 20/50, intraocular pressure (IOP) of 42 mmHg on 0 IOP-lowering medications, and neovascularization of the iris and angle with no peripheral anterior synechiae. Fundoscopy revealed midperipheral dot-blot hemorrhages without diabetic retinopathy or vein occlusion. Fluorescein angiography revealed peripheral retinal nonperfusion in both eyes. The patient was diagnosed with open-angle NVG secondary to OIS and treated with 6 serial monthly anti-VEGF injections interspersed with 4 PRP sessions, after which his anterior segment neovascularization regressed and IOP normalized on 0 medications. Ten weeks after the last injection, the anterior segment neovascularization and elevated IOP recurred, so he underwent 4 more monthly anti-VEGF injections and 4 PRP sessions, after which his anterior segment neovascularization regressed and his IOP normalized on 0 medications. However, 6 weeks after the last injection, the anterior segment neovascularization and elevated IOP again recurred, so he was resumed on a third course of lifetime monthly anti-VEGF injections, which may be continued in perpetuity.

**Conclusion:**

The patient's NVG was quiescent while under the protection of serial anti-VEGF injections with interspersed PRP; however, the disease recurred each time injections were stopped. Therefore, in patients with open-angle NVG secondary to OIS, serial monthly anti-VEGF injections may be necessary combined with PRP to suppress underlying neovascular drive and regress anterior segment neovascularization, maintain physiologic IOP, and prevent synechial angle closure.

## 1. Introduction

Neovascular glaucoma (NVG) is characterized by neovascular proliferation in the anterior segment, specifically the angle (NVA) and iris (NVI), that obstructs aqueous outflow through the trabecular meshwork and closes the angle. Intraocular pressure (IOP) can become profoundly elevated and visual outcomes can be devastating [1]. The underlying etiologies for NVG are conditions that cause retinal ischemia, the two most common being proliferative diabetic retinopathy (PDR) and retinal vein occlusion (RVO) [2]. The third most common etiology is ocular ischemic syndrome (OIS), which is often caused by ipsilateral carotid artery stenosis leading to decreased ocular perfusion [3].

For NVG secondary to PDR or RVO, panretinal photocoagulation (PRP) has been the gold standard treatment to reduce angiogenic signals from the peripheral retina and regress anterior segment neovascularization [4]. In recent years, antivascular endothelial growth factor (VEGF) injections have been shown to promptly regress anterior segment neovascularization within days of administration and have been combined with PRP to treat NVG from PDR and RVO [5–9]. However, there is a paucity of data regarding the therapeutic effects of anti-VEGF and/or PRP in NVG specifically secondary to OIS.

This case report of a patient with NVG secondary to OIS describes the novel strategy of administering a planned series of 6 monthly anti-VEGF injections with multiple sessions of PRP scheduled in between the injections, so the anti-VEGF injections can provide prompt and sustained antineovascular effect for 6 months until full dense PRP can be performed and take effect. We term this treatment protocol the Salvaging Conventional Outflow Pathway in Neovascular Glaucoma (SCOPING) Protocol, and this is our (MQ and DS) standard of care for all NVG patients presenting with partially or completely open angles.

## 2. Case Presentation

**Figure 1:**
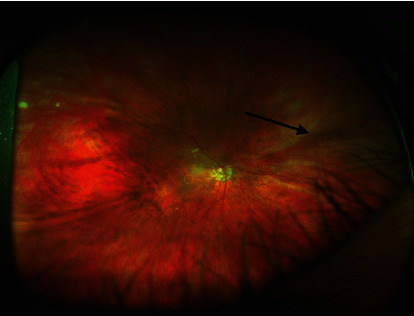
Optos fundus photo shows midperipheral retinal hemorrhages in the right eye.

A 66-year-old White man with a history of hyperlipidemia, hypertension, and coronary artery disease with angioplasty and stenting presented to the ophthalmology clinic for a routine eye exam. The right eye (OD) had a history of pars plana vitrectomy for epiretinal membrane 3 years prior and subsequent uncomplicated cataract surgery 6 months afterward. He used no ocular medications at baseline. He reported no visual complaints, pain, discomfort, photophobia, redness, or headache. His visual acuity (VA) was 20/50 OD and 20/20 OS with -6.5 diopter glasses. The IOP was 47 mmHg OD and 15 mmHg OS. The anterior segment exam OD revealed a clear cornea without microcystic edema, a deep and quiet anterior chamber without hyphema, NVI at the pupil margin in multiple locations, NVA throughout an otherwise open angle with no peripheral anterior synechiae (PAS), and a 1 piece PCIOL in the capsular bag with mild posterior capsule opacity (PCO). The anterior segment exam OS was unremarkable with a clear cornea, deep and quiet anterior chamber, no NVI or NVA, and 2+ nuclear sclerosis. The fundus exam of both eyes revealed tilted myopic nerves with a symmetric cup-to-disc ratio of 0.5 in both eyes and a full neuroretinal rim for 360 degrees in both eyes with no focal rim changes that would suggest glaucomatous optic neuropathy in either eye. There was no macular edema, no exudates, no flame-shaped hemorrhages, attenuated but nontortuous retinal vasculature without visible neovascularization at the disc (NVD) or elsewhere (NVE), and mid-peripheral dot blot hemorrhages (DBH) in both eyes (Figure 1). The fundus exam was not consistent with PDR or RVO, and OIS was suspected.

**Figure 2:**
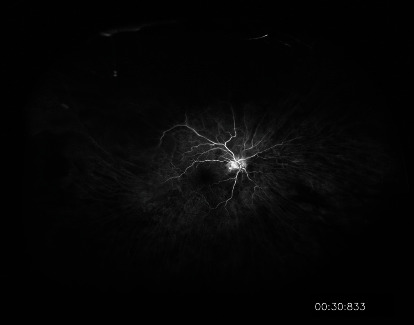
Fluorescein angiography at 30 seconds shows peripheral nonperfusion and prolonged filling time.

The glaucoma service was consulted, and the patient received 3 rounds of timolol, dorzolamide, and brimonidine in the right eye and 500 mg oral acetazolamide; the IOP improved to 16 mmHg 2 hours later. The retina service was consulted, and fluorescein angiography demonstrated profound peripheral retinal non-perfusion in both eyes with no NVD or NVE (Figure 2). The patient was diagnosed with open-angle NVG secondary to OIS in the right eye and underwent prompt intravitreal injection with 1.25 mg (0.05 ml) bevacizumab (IVB) that day.

The antineovascular branch of the treatment plan with the retina service (DS) was to administer at least 6 monthly IVB injections, with multiple sessions of PRP (PASCAL argon laser, Retina 200 lens, parameters described in Table 1) scheduled in between the IVB injections, until the PRP was deemed to be complete. At that point, the IVB injections would be stopped, and the glaucoma service would monitor him for any recurrent anterior segment neovascularization or elevated IOP.

The IOP-control branch of the treatment plan with the glaucoma service (MQ) was to initiate 3 topical IOP-lowering medications (dorzolamide-timolol and brimonidine) and subsequently escalate medical therapy if needed. If the IOP were to become uncontrolled despite maximum tolerated medical therapy and the anterior segment neovascularization was fully regressed at that time, then an angle-based procedure such as gonioscopy-assisted transluminal trabeculotomy would be offered in an attempt to surgically salvage the conventional outflow pathway (consistent with SCOPING Protocol goals) and avoid or delay an aqueous shunt or cyclophotocoagulation, if possible.

A systemic work-up to identify the source of the OIS was performed by the vascular neurology service. A computed tomography angiography demonstrated atherosclerotic calcifications along the bilateral petrous, cavernous, and supraclinoid internal carotid artery (ICA) segments, with multifocal mild and moderate stenoses most pronounced along the bilateral supraclinoid ICA segments. However, the patient was not recommended to pursue neurovascular intervention.

Between Week 0 and Week 22 inclusive, the patient underwent the SCOPING protocol with 6 serial monthly IVB injections interspersed with 4 PRP sessions (Table 1). One day after IVB#1, the visual acuity was still 20/50, the IOP was down from 42 mmHg to 19 mmHg on two IOP-lowering medications, and gonioscopy revealed regressing NVA and no PAS. Throughout the treatment course, the IOP remained physiologic in the teens, even without any IOP-lowering medications after Week 5. He underwent laser capsulotomy at Week 10 for visually significant PCO. At Week 24, two weeks after the SCOPING treatment series, the visual acuity had improved to 20/20, the IOP was 19 mmHg, and gonioscopy revealed fully regressed NVA and no PAS. At this point, his conventional outflow pathway was considered to be “medically salvaged,” and no further antineovascular treatment was recommended by the retina service. He was counseled to continue follow-up with the glaucoma service in 6 weeks.

When the patient presented for follow-up at Week 30, the IOP had risen to 22 mmHg, and gonioscopy revealed recurrent trace NVA in all quadrants and no PAS. He was diagnosed with recurrent anterior segment neovascularization in the setting of stopping serial anti-VEGF injections and underwent a second course of treatment consisting of 4 monthly IVBs and 4 PRP sessions. Only 4 IVBs were planned for this second course rather than 6 because the retina service considered this recurrence to be less severe than the initial presentation. At Week 34, the visual acuity was stable at 20/20, and the IOP was 16 mmHg on no IOP-lowering medications. At Week 38, the IOP was down to 10 mmHg. On Week 48, two weeks after his second course of treatment, the visual acuity was 20/20, the IOP was 20 mmHg, and there was only trace regressing NVA in 2 quadrants on gonioscopy. His conventional outflow pathway was still considered to be “medically salvaged,” in line with the goals of the SCOPING protocol.

When the patient followed up four weeks later at Week 52, the IOP was elevated at 27 mmHg with new NVI and NVA. Considering the repeated recurrent anterior segment neovascularization with elevated IOP, despite a total of 10 IVB injections and more than 4000 total spots of PRP over the span of 52 weeks, the retina service recommended a third “course” of treatment, this time with serial monthly IVB injections in perpetuity (Table 1). Two weeks after IVB#1 of this third series, his IOP was 14 mmHg on one IOP-lowering medication, and the NVI and NVA had regressed yet again. The patient's timolol was stopped, and he was recommended to follow-up with retina monthly in perpetuity for serial monthly IVB injections. At the next retina follow-up visit at Week 56, which is his most recent follow-up to date, the VA OD was still 20/20, and the IOP OD was 13 on 0 IOP-lowering medications. The patient underwent the next IVB injection as planned, and his conventional outflow pathway is still considered to be “medically salvaged.”

## 3. Discussion

For decades, PRP has been the mainstay treatment for NVG, as it has been shown to reverse anterior segment neovascularization and bring elevated IOP levels back to baseline if the angle is not already completely synechially closed [4]. More recently, anti-VEGF injections, including bevacizumab, have been shown to provide rapid-onset antineovascular effects, including regression of anterior segment neovascularization and reduction of IOP if the angle is not already synechially closed [10–12]. However, there is a paucity of literature regarding the treatment of NVG specifically secondary to OIS using PRP and/or anti-VEGF injections. One paper reports that PRP might be viable for patients with neovascularization secondary to common carotid artery occlusion, and another reports that IVB rapidly reduces NVI in patients with OIS [13, 14].

Furthermore, OIS complicates otherwise well-established treatments for NVG. Only 36% of patients with OIS and open angles have regressed NVI and IOP control after PRP [15]. One study did not find retinal capillary dropout in any eyes with OIS, even in patients with diabetes mellitus. Because FA-proven retinal capillary dropout prompts the usage of PRP in cases of NVG secondary to PDR, this suggests that PRP may not always be indicated in cases of NVG secondary to OIS [16]. However, in OIS eyes with FA-proven retinal non-perfusion, PRP is still the preferred treatment [17].

Recently, there have been many studies in the literature regarding using a combination of PRP and anti-VEGF injections for treating NVG. In a study by Ehlers et al., all 11 patients who received same-day IVB and PRP had regressed neovascularization, whereas only 2 of 12 patients who received PRP-alone had regressed neovascularization at an average follow-up of 118 and 143 days, respectively (*p* < 0.001) [5]. The combination group also had better IOP control at follow-up (*p* < 0.005). Of these patients, 3 in the combination group and 4 in the PRP-alone group had OIS. Another retrospective case series by Vasudev et al. reports similar results, comparing 14 eyes that received PRP within 1 week of IVB and 15 that received PRP-alone [7]. At 1-month follow-up, the combination group had more regression of NVI than the PRP-alone group (*p* < 0.005), and at 6-month follow-up, the combination group had a significantly lower IOP than the PRP-alone group (*p* < 0.05). In this study, only 1 eye in the combination group had OIS. Other studies utilizing a combination of PRP and anti-VEGF have also reported prompt regression of NVI and IOP control [6, 8, 9]. To date, there are no studies in the literature focused on the combination of PRP and anti-VEGF in treating NVG secondary to OIS, especially not any studies that stratify outcomes by angle status.

In this report, we describe our strategy of treating NVG secondary to OIS with 2 courses of serial monthly IVB adjuncted with numerous PRP sessions. The quick regression of this patient's anterior segment neovascularization is likely due to the rapid mechanism-of-action of bevacizumab that has previously been reported. The patient's IOP promptly improved from 42 mmHg on 0 IOP-lowering medications on the day of the acute presentation to 19 mmHg on 0 IOP-lowering medications 1 day after the first injection. Out of an abundance of caution, the patient was kept on 2-3 classes of IOP-lowering medications for the first 5 weeks, but it is likely that his IOP would have remained physiologic in this first month even without any IOP-lowering medications, since the therapeutic effects of each IVB lasts approximately 1 month.

Of note, even after multiple sessions of PRP totaling over 4000 spots, when the IVBs were halted for more than 4 weeks after the first and second course of the SCOPING treatment protocol, there was recurrence of anterior segment neovascularization and elevated IOP. Each time, resumption of IVBs rapidly normalized the IOP and induced rapid regression of the anterior segment neovascularization. Fortunately, no PAS developed, and the angle still remained 100% open during this time frame. This case suggests that full dense PRP in the setting of his OIS was not sufficient to completely and permanently suppress the underlying neovascular drive, and he may require ongoing serial monthly IVBs in perpetuity to keep the disease quiescent. Our observations suggest that in patients with NVG secondary to OIS (in contrast to PDR and RVO), regression of neovascularization and IOP control may largely depend on serial anti-VEGF injections, and full dense PRP may not be adequately effective. This conclusion also depends on the angle being mostly open upon presentation, because if the angle were already mostly synechially closed upon presentation, then anti-neovascular treatments, whether it be PRP or anti-VEGF injections, would not be expected to lower the IOP.

There are numerous limitations to this case report. This patient is scheduled to undergo serial monthly IVB in perpetuity, so follow-up exams including monitoring for anterior segment neovascularization and elevated IOP are needed to determine long-term effectiveness of this treatment strategy. Additionally, future studies are needed with larger treatment groups to confirm our findings, but this can be challenging due to the relatively rare nature of NVG secondary to OIS. Importantly, this patient presented with a 100% open angle, so all findings in this report may only be pertinent to patients with open-angle NVG secondary to OIS. More evidence is needed regarding the best treatment strategy in eyes with NVG secondary to OIS presenting with angles that are already partially or totally synechially closed. This highlights the need for an updated standardized nomenclature for various stages of NVG to help clinicians and researchers better categorize their broad spectrum of “NVG” patients for both research trials and clinical practice [18].

In conclusion, antineovascular treatment plans combining prompt serial monthly anti-VEGF injections with interspersed PRP can rapidly reverse anterior segment neovascularization and may provide long-term IOP control and prevent progressive synechial angle closure in patients with open-angle NVG secondary to OIS. This may prove to be an effective alternative for patients that have previously undergone PRP-alone without sustained success. If there is a planned hiatus of more than 4 weeks between antineovascular treatments with the retina service, close follow-up is recommended with an anterior segment service (glaucoma, comprehensive) to monitor for recurrent anterior segment neovascularization and elevated IOP in the absence of serial monthly IVBs in perpetuity.

## Figures and Tables

**Table 1:** Details of antineovascular and intraocular pressure treatment course of the right eye.

Weeks after presentation	Treatment	IOP (mmHg)	# Meds	Service(s)
First Course				
0	IVB #1	42	0	O‐>G‐>R
1	None	9	3	G
4	IVB #2	10	2	R
5	None	10	2	G
6	PRP #1 (1118 spots, 225 mW)	19	0	R
8	IVB #3	19	0	R
10	PRP #2 (596 spots, 275 mW)	21	0	R
12	IVB #4	17	0	R
14	PRP #3 (193 spots, 225 mW)	N/A	0	R
16	IVB #5	14	0	R
20	IVB #6	15	0	R
22	PRP #4 (462 spots, 200 mW)	N/A	0	R
24	None	19	0	G
Second Course				
30	IVB #1	22	0	G‐>R
31	PRP #1 (322 spots, 375 mW)	16	0	R
34	IVB #2	16	0	R
35	PRP #2 (411 spots, 200 mW)	13	0	R
38	IVB #3	10	0	R
40	PRP #3 (560 spots, 200 mW)	N/A	0	R
42	IVB #4	18	0	R
46	PRP #4 (866 spots, 200 mW)	22	0	R
48	None	20	0	G
Third Course				
52	IVB #1	27	0	R
54	None	14	1	G
56	IVB #2	13	0	R
Future	Ongoing serial monthly IVB			

IVB: intravitreal bevacizumab 1.25 mg in 0.05 ml; PRP: panretinal photocoagulation; O: optometry; G: glaucoma; R: retina spot size for PRP was 400 microns in the first course of treatment and 200 microns in the 2nd course of treatment. Duration was 0.5-0.7 seconds.

## Data Availability

All data used to support the findings of this study are included within the article.
